# Circular RNA cMTO1 Promotes PTEN Expression Through Sponging miR-181b-5p in Liver Fibrosis

**DOI:** 10.3389/fcell.2020.00714

**Published:** 2020-07-31

**Authors:** Hui Jin, Chunxue Li, Peihong Dong, Junting Huang, Jinglu Yu, Jianjian Zheng

**Affiliations:** ^1^Department of Pharmacy, The First Affiliated Hospital of Wenzhou Medical University, Wenzhou, China; ^2^Key Laboratory of Diagnosis and Treatment of Severe Hepato-Pancreatic Diseases of Zhejiang Province, The First Affiliated Hospital of Wenzhou Medical University, Wenzhou, China; ^3^Department of Infectious Diseases, The First Affiliated Hospital of Wenzhou Medical University, Wenzhou, China; ^4^Department of Laboratory Medicine, Lishui Municipal Central Hospital, Lishui, China

**Keywords:** cMTO1, miR-181b-5p, liver fibrosis, hepatic stellate cell, PTEN

## Abstract

**Background:**

Circular RNAs (circRNAs) are considered as key regulators of cancer biology. Recently, cMTO1 (a circRNA derived from MTO1 gene, hsa_circ_0007874) has been demonstrated to act as a tumor suppressor in hepatocellular carcinoma (HCC). However, the roles of cMTO1 in liver fibrosis are largely unknown.

**Methods:**

Expressions and roles of cMTO1 were examined *in vivo* and *in vitro* during liver fibrosis. The interaction between microRNA-181b-5p (miR-181b-5p) and cMTO1 was analyzed by luciferase activity assays and pull down assays.

**Results:**

cMTO1 was shown to be reduced in the liver from patients with cirrhosis. In addition, cMTO1 was down-regulated in the mouse fibrotic livers as well as activated hepatic stellate cells (HSCs). Restoring of cMTO1 led to a reduction in HSC proliferation. Results of immunofluorescence analysis showed that cMTO1 suppressed the expressions of α-SMA and type I collagen. cMTO1 was found to be expressed in the cytoplasm of HSCs. Further studies confirmed that cMTO1 and miR-181b-5p were co-located in the cytoplasm. Interestingly, there was an interaction between cMTO1 and miR-181b-5p. Results of luciferase reporter assays and pull down assays confirmed that miR-181b-5p could bind to cMTO1. cMTO1-inhibited HSC activation was blocked down by miR-181b-5p or PTEN. Meanwhile, PTEN was a target of miR-181b-5p.

**Conclusion:**

cMTO1 inhibits HSC activation, at least in part, through miR-181b-5p-mediated PTEN expression. Our results also suggest that cMTO1 may be a novel therapeutic target in liver fibrosis.

## Introduction

Liver fibrosis is a dynamic process characterized by the net accumulation of extracellular matrix components, especially collagens (Friedman, 2008). MicroRNAs (miRNAs) are a class of endogenous small non-coding RNAs molecules that regulate gene expression by mRNA degradation and/or translation repression (Croce and Calin, 2005). miRNAs have been reported to be deregulated in various human diseases and involved in many biological processes such as cell proliferation (Ma et al., 2018; Bruch et al., 2019). Currently, a growing body of evidence suggests that miRNAs participate in the control of hepatic stellate cell (HSC) activation, which is an essential event in liver fibrosis (Kim et al., 2019). We previously found that miR-17-5p, up-regulated in activated HSCs, promotes HSC activation via targeting of smad7 (Yu et al., 2015). Therefore, miRNAs are vital HSC regulators as well as promising therapeutic targets in liver fibrosis.

Circular RNAs (circRNAs) represent an abundant class of non-coding RNAs in mammalian cells and are characterized by covalently closed loop structures without a free 3′ or 5′ end (Hentze and Preiss, 2013; Chen and Yang, 2015). Due to the special structure of circRNAs, they are inherently resistant to exonucleoltytic RNA decay. CircRNAs, as potential biomarkers of diseases, are found to exhibit species conservation and high tissue-specific expression (Memczak et al., 2013). Increasing evidence has shown that dysregulation of circRNAs could be found in various cancers including bladder cancer, gastric cancer and hepatocellular carcinoma (HCC) (Wang et al., 2017; Yang et al., 2018; Yu et al., 2018). CircRNAs are also implicated in the regulation of many biological processes such as proliferation and differentiation (Zhao and Shen, 2017). Emerging evidence has revealed that circRNAs could sponge miRNAs in many cancers by acting as competing endogenous RNAs and thereby lead to the derepression of miRNA targets (Deng et al., 2018; Yu et al., 2018). However, to our knowledge to date, little is known about the roles of circRNAs in the progression of liver fibrosis.

Recently, cMTO1 (a circRNA derived from MTO1 (mitochondrial tRNA translation optimization 1) gene, hsa_circ_0007874) has been demonstrated to be a tumor suppressor in HCC (Han et al., 2017). cMTO1 is located at chr6:74175931-74176329 (circBase database: http://www.circbase.org/cgi-bin/simplesearch.cgi). In addition, Han et al. (2017) demonstrated that cMTO1 is down-regulated in HCC, which is associated with HCC prognosis. However, the roles of cMTO1 in liver fibrosis remain unclear.

## Materials and Methods

### Materials

Chemically synthesized RNAs including negative control (miR-NC), miR-181b-5p mimics and inhibitor were obtained from GenePharma biotechnology (Shanghai, China). For transfection, the cells were transfected with 1 μg of the chemically synthesized RNA. Adenoviral vectors expressing cMTO1 (Ad-cMTO1), adenoviral vectors expressing a control scrambled sequence (Ad-Ctrl), adenoviral vectors expressing shRNA against cMTO1 (Ad-shcMTO1) and adenoviral vectors expressing the scrambled shRNA (Ad-shCtrl) were purchased from GenePharma biotechnology.

### Clinical Samples

In this study, 35 healthy controls and 35 liver cirrhosis patients undergoing partial liver resection or liver biopsy were selected from the First Affiliated Hospital of Wenzhou Medical University in Wenzhou from 2011.1 to 2013.6 (Table 1). Liver cirrhosis was diagnosed by liver biopsy and/or a typical appearance of the liver on abdominal ultrasound and/or computed tomography scan. Informed consent for use of liver samples was obtained from all participants. The project was approved by the Ethics Committee of the First Affiliated Hospital of Wenzhou Medical University (Wenzhou, China) and all procedures were performed in compliance with the Declaration of Helsinki.

**TABLE 1:** Human subjects.

**Parameters**	**Patients**	**Controls**
Cases, *n*	35	35
**Sex, *n* (%)**		
Male	18 (51.4%)	19 (54.2%)
Female	17 (48.6%)	16 (45.8%)
Age, *n* (±SD)	45.2 (±5.5)	46.1 (±4.1)
**Etiology, *n* (%)**		
HBV	35 (100%)	−
Serum ALT, U/L	115 (±35.4)	30.5 (±8.5)
Serum AST, U/L	125.6 (±26.2)	22.2 (±4.1)
**Child-Pugh stage**		
A	16 (45.7%)	−
B	11 (31.4%)	−
C	8 (22.9%)	−

### Carbon Tetrachloride (CCl_4_) Liver Injury Model

To generate liver fibrosis, eight-week-old male C57BL/6J mice (*n* = 6) received an intraperitoneal injection of 7 μL/g of 10% CCl_4_ (Sigma-Aldrich, St. Louis, MO, United States) in olive oil two times weekly for 8 weeks. Moreover, the control mice (*n* = 6) were treated with olive oil with equal volumes at the same time intervals. Mice were sacrificed under anesthesia after CCl_4_ treatment. All mice were provided by the Experimental Animal Center of Wenzhou Medical University. All procedures were approved by the Animal Ethics Committee of Wenzhou Medical University. A part of the liver tissues were fixed, embedded in paraffin, and processed for Masson staining. Quantitative analysis for the Masson-positive area was calculated from five fields for each liver slice. Other liver tissues were stored at −80°C for further analysis.

### Hepatic Hydroxyproline Content

Liver tissues (50 mg) were homogenized in HCl and hydrolyzed at 120°C overnight. After lysate centrifugation at 12,000 × *g* for 10 min at 4°C, the supernatant was evaporated to dryness under vacuum. The hepatic hydroxyproline content was assessed using the Hydroxyproline Colorimetric Assay kit (BioVision, San Francisco, CA, United States). Data were normalized to liver weight.

### Isolation and Culture of Primary HSCs

Primary HSCs were isolated as described previously (Chang et al., 2014). The isolated cells were seeded in tissue culture plates and cultured in DMEM with 10% fetal bovine serum, 100 U/mL penicillin, and 100 μg/mL streptomycin. The purity of cultures was confirmed by immunocytochemical staining for α-smooth muscle actin (α-SMA) and the purity reached > 98%.

### Quantitative Real-Time PCR (qRT-PCR)

Total RNA was extracted from cells and liver tissues using the miRNeasy Mini Kit (Qiagen, Valencia, CA, United States). Fifty nanograms RNA was reverse-transcribed to cDNA using the ReverTra Ace qPCR RT Kit (Toyobo, Osaka, Japan) in accordance with the manufacturer’s instructions. Cytoplasmic or nuclear cMTO1 was isolated from HSCs using cytoplasmic and nuclear RNA purification kits (Norgen, Thorold, Canada) as detailed previously (Zaghlool et al., 2013). Gene expression was measured by real-time PCR using cDNA, SYBR Green real-time PCR Master Mix (Toyobo, Osaka, Japan), and a set of gene-specific oligonucleotide primers (Table 2). The primers of alpha-1(I) collagen (Col1A1), α-SMA, phosphatase and tensin homolog (PTEN), GAPDH and hsa-cMTO1 were designed as described previously (Han et al., 2017; Yu et al., 2017; Geng et al., 2020). TaqMan MicroRNA Assays (Applied Biosystems, Foster City, CA, United States) were performed to detect 42 miRNAs expressions. The GAPDH (Applied Biosystems, Foster City, CA, United States) level was used to normalize the relative abundance of cMTO1 and mRNAs. U6 snRNA (Applied Biosystems, Foster City, CA, United States) was used to normalize the relative abundance of miRNAs. The expression levels (2^–ΔΔCt^) of cMTO1, mRNAs, and miRNAs were calculated.

**TABLE 2:** Primer sequences.

**Gene**	**Forward sequence**	**Reverse sequence**
mmu_circ_0015909	5′-GGAACCACTGGCTATGAGGA-3′	5′-GGATGCATCTCCAATCCAAT-3′
mmu_circ_0015910	5′-GCAGAGCAGACAGCAGTGAC-3′	5′-GGATGCATCTCCAATCCAAT-3′
mmu_circ_0015911	5′-CCCGCAAGGGTTATCTGTTA-3′	5′-AAGGACAATCGCATCCACTC-3′
mmu_circ_0015912	5′-CAGTCCTTCCCTCGAGACAC-3′	5′-GGGGTTGGTGTGAGTCAAGT-3′
mmu_circ_0015913	5′-CAGTGAGCCATACCGAATGTT-3′	5′-TGAACGTGGCTATTCAGGTG-3′
mmu_circ_0015914	5′-CCGAGCCCTTGGAGAAGTAT-3′	5′-AAGGACAATCGCATCCACTC-3′
mmu_circ_0015915	5′-CCATCAGGGATGTGTCATTG-3′	5′-TGAACGTGGCTATTCAGGTG-3′

### Western Blot Analysis

Tissues and cells were lysed with ice-cold lysis buffer (50 mM Tris–HCl, pH 7.4, 100 mM 2-Mercaptoethanol, 2% w/v SDS, 10% glycerol). Proteins were subjected to SDS-PAGE and transferred to PVDF membranes (Millipore, Billerica, MA, United States). After blocking, membranes were incubated with primary antibodies against PTEN and GAPDH (Abcam, Cambridge, MA, United States) overnight at 4 °C, followed by secondary IRdye 800-conjugated goat anti-mouse IgG or goat anti-rabbit IgG (Rockland, Limerick, PA, United States) at 37 °C for 1 h. The levels of protein were normalized to total GAPDH.

### 5-Ethyny-2′-Deoxyuridine (EdU) Assays

After treatment with Ad-cMTO1 or Ad-shcMTO1, HSCs were labeled with EdU for 12 h. HSC proliferative rate was detected using a Cell-Light EdU *In Vitro* Imaging Detection Kit (RiboBio Co., Ltd., Guangzhou, China) according to the manufacturer’s instructions.

### Immunofluorescence Microscopy

Cells were seeded on 18-mm cover glasses and fixed in an acetic acid: ethanol (1:3) solution for 5 min at −20°C. 5% goat serum/PBS was used to block nonspecific binding for 1 h at room temperature. Next, cells were incubated with primary antibodies against α-SMA or type I collagen, followed by fluorescein-labeled secondary antibody (1:50 dilution; Dianova). The nuclei were stained with 4,6-diamidino-2-phenylindole (DAPI). The slides were washed twice with PBS, covered with DABCO (Sigma-Aldrich), and examined with confocal laser scanning microscopy (Olympus, Tokyo, Japan) at 568 nm.

### Fluorescence *in situ* Hybridization (FISH)

The double FISH assay was performed in cells as previously described (Yu et al., 2018), with minor modifications. Biotin-labeled probes specific to cMTO1 and Dig-labeled miR-181b-5p probes were used in the hybridization. The signals of biotin-labeled probes were detected using Cy5-Streptavidin (Life Technologies). The signals of Dig-labeled miR-181b-5p probes were detected using a tyramide-conjugated Alexa 488 fluorochrome TSA kit. Nuclei were counter-stained with DAPI. Images were acquired on a Leica TCS SP2 AOBS confocal microscope (Leica Microsystems, Mannheim, Germany).

### RNA Binding Protein Immunoprecipitation (RIP) Assay

RIP experiment was conducted using the EZ-Magna RIP Kit (Millipore) according to the manufacturer’s instructions. Briefly, primary HSCs at 80–90% confluency were lysed in complete RIP lysis buffer, followed by incubation with RIP buffer including magnetic beads coupled with anti-Argonaute-2 (Ago2) antibody (Abcam). Isotype-matched immunoglobulin G (IgG) was used as a negative control. After samples were incubated with proteinase k, immunoprecipitated RNA was isolated. qRT-PCR was performed to analyze cMTO1 level in the precipitates.

### Pull-Down Assay With Biotinylated miR-181b-5p (Bio-miR-181b-5p)

Biotin pull-down was performed as previously described (Wang et al., 2014). Briefly, after 48 h of HSCs transfected with Bio-miR-181b-5p-Wt, Bio-miR-181b-5p-Mut, or Bio-miR-NC, the cells were washed with PBS followed by incubation in a lysis buffer for 10 min. To exclude RNA and protein complexes, the beads were blocked in lysis buffer including RNase-free bovine serum albumin and yeast tRNA (Sigma). After the lysates were incubated with streptavidin-coated magnetic beads (Life Technologies) at 4°C for 4 h, they were washed twice with lysis buffer, three times with the low salt buffer, and once with the high salt buffer. The bound RNAs were isolated using TRIzol reagent (Life Technologies). cMTO1 expression was determined by qRT-PCR.

### Dual Luciferase Reporter Assay

pmirGLO-cMTO1 was cotransfected with the predicted miRNAs or miR-NC into HEK293T cells by lipofectamine-mediated gene transfer as described previously (Yu et al., 2017). The relative luciferase activity was normalized to Renilla luciferase activity 48 h after transfection (Invitrogen).

### Statistical Analysis

Data from at least three independent experiments were expressed as the mean ± SD. Differences between multiple groups were evaluated using one-way analysis of variance. Differences between two groups were compared using a Student’s *t*-test. The Mann-Whitney test was performed to determine the significance of liver cMTO1 levels. Pearson’s test was used for the correlation analysis between two groups. Receiver operating characteristic (ROC) curve was generated to evaluate the diagnostic potential of cMTO1 via calculation of the area under the ROC curve (AUC), sensitivity and specificity according to the standard formulas. *P* < 0.05 was considered significant. All statistical analyses were performed with SPSS software (version 13; SPSS, Chicago, IL, United States).

## Results

### Low Expression of cMTO1 in the Livers From Patients With Cirrhosis

**FIGURE 1:**
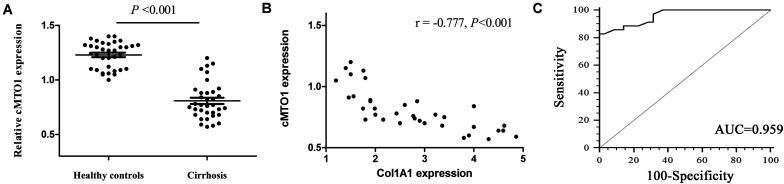
Expression of cMTO1 in cirrhotic human liver tissues. **(A)** Lower cMTO1 expression was found in patients with cirrhosis (*n* = 35) compared with that in healthy controls (*n* = 35). **(B)** Negative correlation between transcriptional level of Col1A1 and cMTO1 in the livers from patients with cirrhosis. **(C)** ROC analysis of cMTO1 for discriminating patients with cirrhosis from healthy controls.

To determine whether cMTO1 exerts an effect on liver fibrosis, the liver cMTO1 expression in patients with cirrhosis was firstly detected. In comparison with the healthy control group, lower cMTO1 expression was found in the cirrhosis group (Figure 1A). Next, the correlation between cMTO1 expression and Col1A1 mRNA level in cirrhotic liver tissues was investigated. The correlational analysis showed that cMTO1 expression was negatively correlated with the transcriptional level of Col1A1 (*r* = −0.777, *P* < 0.001, Figure 1B). We additionally analyzed the potential diagnostic value of cMTO1 for liver fibrosis. Using ROC curve analysis, liver cMTO1 differentiated liver cirrhosis patients from healthy controls, with an AUC of 0.959 [95% confidence interval (CI), 0.883 to 0.992] (Figure 1C). Moreover, its sensitivity was 82.8% as well as its specificity was 100% when the cutoff value was 0.92. Our data suggest the possible involvement of cMTO1 in liver fibrosis.

### Down-Regulated cMTO1 Is Found *in vivo* and *in vitro* During Liver Fibrosis

**FIGURE 2:**
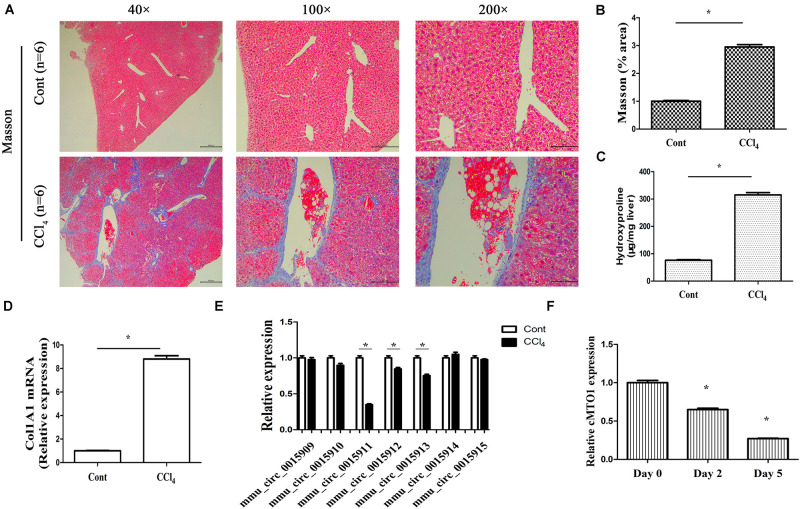
Expression of cMTO1 in CCl_4_-induced hepatic fibrotic tissues as well as primary HSCs. **(A,B)** Masson staining for assessing collagen deposition. Scale bar = 400 μm for 40×, 200 μm for 100× and 100 μm for 200×. The assessment of Masson area was finished at 100×. **(C)** Hydroxyproline level. **(D)** Col1A1 mRNA level. **(E)** circRNA splices derived from mouse MTO1 gene including mmu_circ_0015909, mmu_circ_0015910, mmu_circ_0015911, mmu_circ_0015912, mmu_circ_0015913, mmu_circ_0015914 and mmu_circ_0015915 were detected in CCl_4_ mice. **(F)** Expression of cMTO1 was analyzed in isolated primary HSCs at different culture time. *P < 0.05 compared to the control. Each value is the mean ± SD of three experiments.

One of the prominent features of fibrotic diseases is excessive deposition of collagens. CCl_4_, as a commonly used hepatotoxic reagent, was used to generate a mouse liver fibrosis model. To determine the establishment of mouse liver fibrosis model, the total collagen level was evaluated by both Masson staining and hydroxyproline analysis. The results of Masson staining indicated an obvious increase in collagen expression in the livers of CCl_4_ mice when compared with that in the control mice (Figures 2A,B). Accordingly, liver hydroxyproline content suggested a markedly collagen accumulation in the CCl_4_ group (Figure 2C). CCl_4_ additionally resulted in a significant increase in Col1A1 mRNA level (Figure 2D). These results established the model of CCl_4_ caused-liver fibrosis. In circBase (a database for circRNA, http://www.circbase.org/), there are seven circRNA splices derived from mouse MTO1 gene. They are mmu_circ_0015909, mmu_circ_0015910, mmu_circ_0015911, mmu_circ_0015912, mmu_circ_0015913, mmu_circ_0015914, and mmu_circ_0015915. It was found that the expressions of mmu_circ_0015911, mmu_circ_0015912 and mmu_circ_0015913 were reduced in CCl_4_ mice whereas others not (Figure 2E). Notably, expression of mmu_circ_0015911 was lower than expressions of mmu_circ_0015912 and mmu_circ_0015913. Therefore, mmu_circ_0015911 was selected for the next experiments. It is known that isolated primary mouse HSCs will be gradually activated during culture days. Next, cMTO1 expression was examined in isolated primary HSCs from healthy mice. It was confirmed that cMTO1 was decreased during HSC activation (Figure 2F). All these results suggest that cMTO1 is down-regulated *in vivo* and *in vitro* during liver fibrosis.

### Over-Expression of cMTO1 Reduces Activation of HSCs

**FIGURE 3:**
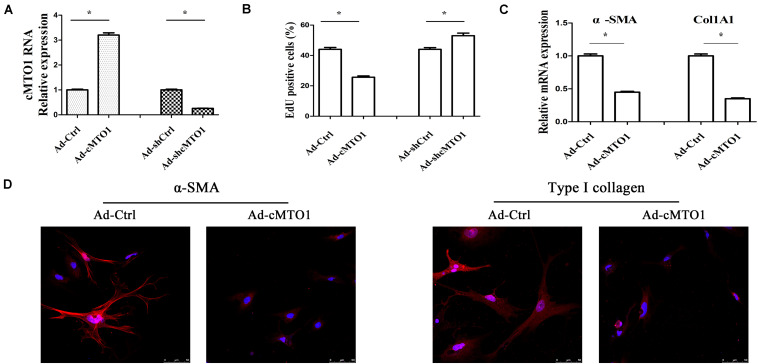
Effects of cMTO1 on HSC activation. Primary 1-day-old HSCs were transduced with Ad-cMTO1 or Ad-shcMTO1 for 48 h. **(A)** cMTO1 expression. **(B)** HSC proliferation. **(C)** The mRNA expression levels of α-SMA and Col1A1. **(D)** Immunofluorescence staining for α-SMA (red) and type I collagen (red) was analyzed in cMTO1-over-expressing HSCs by confocal laser microscopy. DAPI stained nuclei blue. Scale bar = 50 μm for 400×. *P < 0.05. Each value is the mean ± SD of three experiments.

Next, we explored the biological functions of cMTO1 in HSC activation. Due to its low expression during liver fibrosis, cMTO1 was subsequently over-expressed in primary HSCs. As indicated by Figure 3A, Ad-cMTO1 treatment led to a significant increase in cMTO1 expression in HSCs while Ad-shcMTO1 caused a reduction in cMTO1 level. Next, HSC proliferation was evaluated by Edu assays. Our results showed that compared with the control, cell proliferation was down-regulated by cMTO1 over-expression (Figure 3B). In line with it, loss of cMTO1 induced an increase in cell proliferation (Figure 3B). Then, the effect of cMTO1 on HSC transdifferentiation was examined. cMTO1 caused a significant decrease in α-SMA mRNA level (Figure 3C). Fibers of α-SMA were reduced by cMTO1, as indicated by immunofluorescence analysis (Figure 3D). We also assessed the effect of cMTO1 on collagen expression. There was a significant reduction in Col1A1 mRNA level in cMTO1-over-expressing cells (Figure 3C). Also, immunofluorescence analysis indicated a reduction in type I collagen level in cMTO1-over-expressing cells (Figure 3D). All data suggest an anti-fibrotic role of cMTO1 in HSC activation.

### cMTO1 Sponges miR-181b-5p

**FIGURE 4:**
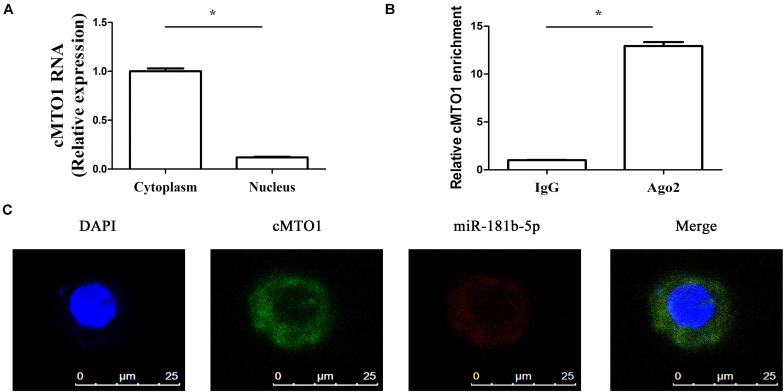
cMTO1 could bind to miRNAs. **(A)** The distribution of cMTO1 in cytoplasm and nucleus of HSCs. **(B)** RIP experiments were performed using Ago2 antibody on extracts from primary HSCs. Relative level of cMTO1 was expressed as fold enrichment in Ago2 relative to IgG immunoprecipitates by qRT-PCR. **(C)** Co-localization between miR-181b-5p and cMTO1 was observed by RNA in situ hybridization. *P < 0.05. Each value is the mean ± SD of three experiments.

**FIGURE 5:**
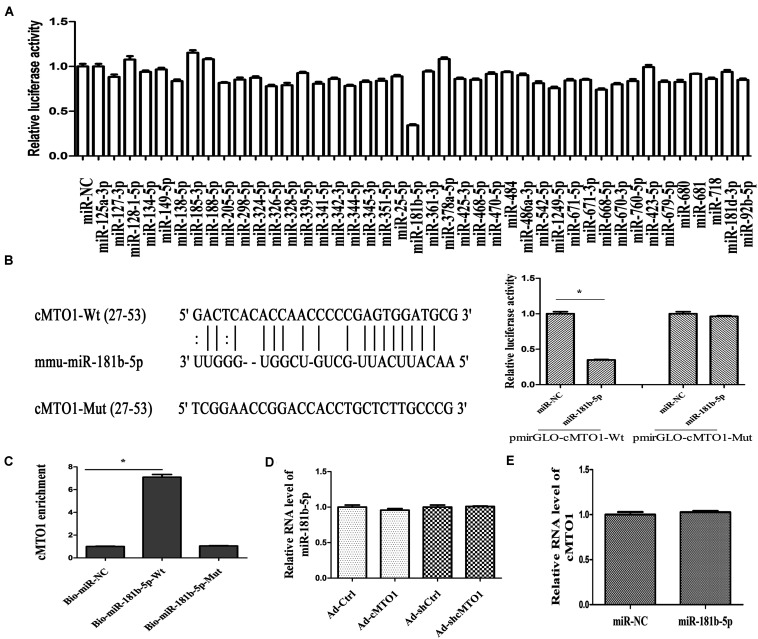
The interaction between cMTO1 and miR-181b-5p. **(A)** The luciferase activity of pmirGLO-cMTO1-Wt in HEK-293T cells after co-transfection with the indicated 42 miRNAs or miR-NC. **(B)** A schematic drawing indicated the putative binding sites of miR-181b-5p with respect to cMTO1. Moreover, relative luciferase activities of pmirGLO-cMTO1-Wt or pmirGLO-cMTO1-Mut were analyzed in HEK-293T cells. **(C)** To validate the direct interaction between cMTO1 and miR-181b-5p, the pull-down assay was performed. Bio-miR-NC is not complementary to cMTO1. **(D)** Expression of miR-181b-5p in HSCs after Ad-cMTO1 or Ad-shcMTO1 treatment for 48 h. **(E)** Expression of cMTO1 in HSCs after miR-181b-5p transfection for 48 h. *P < 0.05. Each value is the mean ± SD of three experiments.

A large body of evidence has shown that many circRNAs serve as sponges for miRNAs, leading to the derepression of miRNA targets (Yu et al., 2018). Particularly, circRNAs that sponge miRNAs are mostly distributed in the cytoplasm. Thus, the distribution of cMTO1 in HSCs was examined by qRT-PCR. Our results indicated the location of cMTO1 in the cytoplasm of HSCs (Figure 4A). In addition, analysis of RIP experiment showed that cMTO1 was enriched by about 13.0-fold in the Ago2 pellets in comparison with that in IgG pellets (Figure 4B). As confirmed by FISH analysis, cMTO1 was mainly distributed in the cytoplasm of HSCs (Figure 4C). Combined with these, cMTO1 may act as a binding platform for miRNAs. Subsequently, we explored the underlying anti-fibrotic mechanisms of cMTO1 in HSC activation. MiRNA target prediction tools (miRanda and RNAhybrid) were used to find potential miRNAs that could bind to cMTO1. Bioinformatic analysis predicted 42 potential miRNAs that may bind to cMTO1 (Figure 5A). Luciferase activity assays were performed to further confirm whether these miRNA recognition sequences on cMTO1 were affected by those potential miRNAs. Compared with miR-NC group, 35 miRNAs were found to reduce luciferase reporter activities. Notably, miR-181b-5p mimics resulted in the lowest luciferase reporter activity among all the miRNAs (Figure 5A). Further studies were performed to confirm the relation between miR-181b-5p and cMTO1. We generated a cMTO1 luciferase reporter containing the miR-181b-5p-mutated sites (Figure 5B). miR-181b-5p led to a significant reduction in luciferase activity of cMTO1-Wt, whereas it had no effect on cMTO1-Mut luciferase activity (Figure 5B). Our data suggest the existence of the interaction between cMTO1 and miR-181b-5p. To further confirm it, a pull down assay was performed using bio-miR-181b-5p. cMTO1 enrichment was increased by bio-miR-181b-5p while there was no significant change caused by bio-miR-181b-5p-Mut (Figure 5C). As indicated by Figure 4C, a co-localization between miR-181b-5p and cMTO1 was confirmed by FISH analysis, suggesting that cMTO1 binds to miR-181b-5p in HSCs. In addition, cMTO1 over-expression or knockdown had no effect on miR-181b-5p expression (Figure 5D). Likewise, miR-181b-5p mimics did not induce a change in cMTO1 expression (Figure 5E).

### cMTO1 Inhibits HSC Activation via miR-181b-5p

**FIGURE 6:**
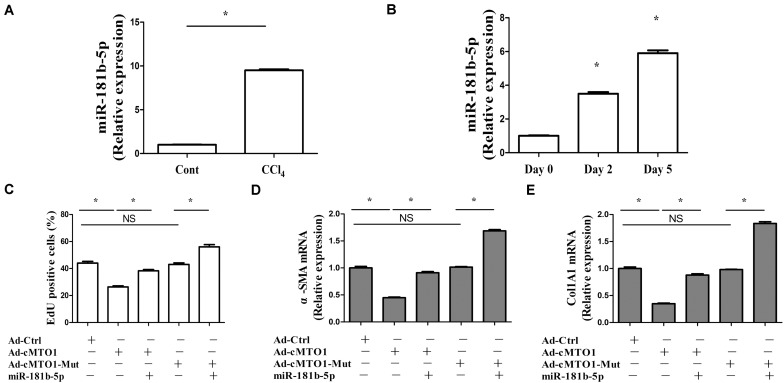
miR-181b-5p takes a part in the effects of cMTO1 on HSC activation. Primary 1-day-old HSCs were transduced with Ad-cMTO1 or Ad-cMTO1-Mut for 48 h and transfected with miR-181b-5p mimics for additional 48 h. **(A)** miR-181b-5p level in CCl_4_ mice. **(B)** miR-181b-5p level in primary HSCs. **(C)** Reduced cell proliferation by cMTO1 over-expression was restored by miR-181b-5p. **(D,E)** cMTO1-suppressed α-SMA and Col1A1 were blocked down by miR-181b-5p. *P < 0.05 compared to the control. Each value is the mean ± SD of three experiments.

In fact, the roles of miR-181b-5p have been explored in liver fibrosis. miR-181b-5p is found to play a pro-fibrotic role in liver fibrosis (Wang et al., 2012; Zheng et al., 2015). Further studies were performed to determine whether miR-181b-5p was involved in the effects of cMTO1 on HSC activation. Next, miR-181b-5p expression was detected in mouse fibrotic liver tissues as well as primary HSCs. miR-181b-5p was found to be increased *in vivo* and *in vitro* during liver fibrosis (Figures 6A,B), which was consistent with the previous studies (Zheng et al., 2015). It was observed that the suppression of HSC proliferation by cMTO1 was blocked down by miR-181b-5p mimics (Figure 6C). Similarly, the inhibition of HSC transdifferentiation induced by cMTO1 was reversed by miR-181b-5p mimics (Figure 6D). Reduced collagen expression caused by cMTO1 was restored by miR-181b-5p mimics (Figure 6E). However, over-expression of cMTO1-Mut caused no effect on HSC activation including proliferation, α-SMA and Col1A1 (Figures 6C–E). In comparison with the cMTO1-Mut group, miR-181b-5p promoted HSC activation (Figures 6C–E), suggesting that miR-181b-5p is a pro-fibrotic regulator in liver fibrosis. All the results suggest that cMTO1 inhibits HSC activation, at least in part, through sponging miR-181b-5p.

### cMTO1 Reduces HSC Activation via miR-181b/PTEN Axis

**FIGURE 7:**
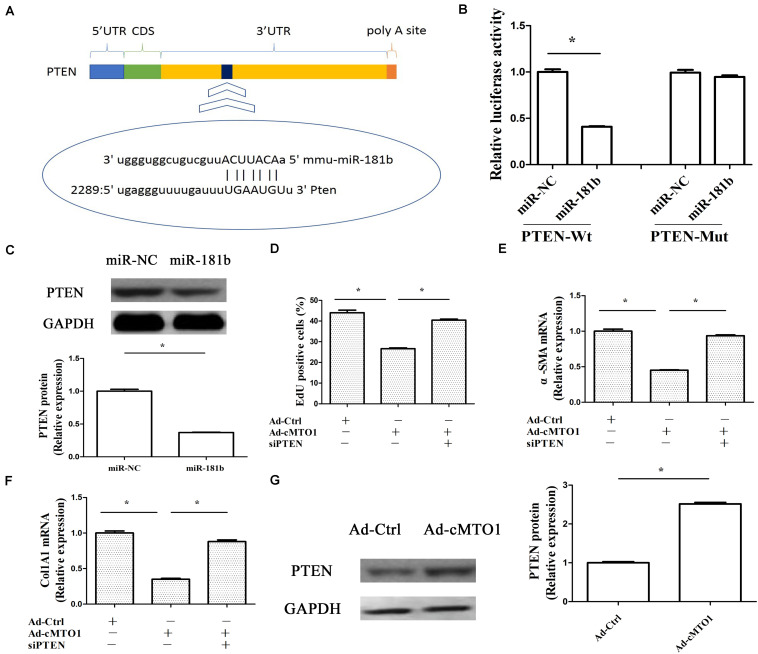
Anti-fibrotic roles of cMTO1 in HSCs were via miR-181b/PTEN axis. Primary 1-day-old HSCs were transduced with Ad-cMTO1 for 48 h and transfected with PTEN siRNA for additional 48 h. **(A)** A schematic drawing indicated the putative binding sites of miR-181b-5p with respect to PTEN. **(B)** Relative luciferase activities of PTEN-Wt or PTEN-Mut were analyzed in HEK-293T cells. **(C)** PTEN protein in miR-181b-transfected cells. **(D)** Cell proliferation down-regulated by cMTO1 was restored by PTEN siRNA. **(E,F)** Loss of PTEN inhibited cMTO1-reduced α-SMA and Col1A1. **(G)** PTEN protein in cMTO1-overexpressing cells. *P < 0.05. Each value is the mean ± SD of three experiments.

PTEN, a negative regulator of HSC activation, has been reported to play an anti-fibrotic role in liver fibrosis via PI3K/Akt pathway (Parsons et al., 2007). Recent studies have shown that miR-181b-mediated PTEN is involved in HSC activation (Geng et al., 2020). Herein, whether miR-181b/PTEN axis participates in the effects of cMTO1 on HSC activation was examined. As shown in Figure 7A, bioinformatic analysis (microRNA.org and miRDB) predicted that PTEN had a potential miR-181b-binding site. Results of luciferase reporter activity assays confirmed that PTEN was a target of miR-181b-5p, with a reduction in PTEN-Wt (Figure 7B). Consistent with it, miR-181b over-expression led to a significant reduction in PTEN protein (Figure 7C). Further studies were performed to explore whether PTEN was involved in the effects of cMTO1 on HSC activation. Our results showed that loss of PTEN inhibited cMTO1-reduced cell proliferation (Figure 7D). Silencing PTEN blocked down the inhibitory effects of cMTO1 on α-SMA and collagen expression (Figures 7E,F). Moreover, over-expression of cMTO1 led to a significant increase in PTEN protein expression (Figure 7G). We also explored the roles of miR-181b in PTEN-dependent HSC activation. Clearly, loss of miR-181b led to an increase in PTEN protein, with a reduction in α-SMA and Col1A1 (Supplementary Figure S1). All these data suggest that cMTO1 reduces HSC activation, at least in part, through miR-181b/PTEN axis.

## Discussion

CircRNAs, a novel class of RNAs with a closed loop structure, are firstly observed in RNA viruses in the 1970s (Wu et al., 2012; Wilusz and Sharp, 2013). Currently, far more than 25,000 circRNAs have been identified in human fibroblasts, and the relative abundance of their expressions may be 10-fold or higher compared to their associated linear mRNAs (Jeck et al., 2013). CircRNAs have highly conserved sequences and a stable existence with a half-life > 48 h (Hansen et al., 2013; Jeck et al., 2013). In addition, circRNAs are shown to be predominantly expressed in the cytoplasm and enriched in exosomes (Li Y. et al., 2015). Recent evidence suggests that desregulation of circRNAs is associated with disease progression and circRNAs can serve as potential biomarkers for the diagnosis and prognosis of cancers (Cui et al., 2016; Zhang et al., 2018). At the present study, it was found that liver cMTO1 was lower in patients with cirrhosis than that in healthy controls. Liver cMTO1 negatively correlated with liver Col1A1 expression, suggesting that cMTO1 may be involved in the progression of liver fibrosis. We also explored whether liver cMTO1 could be a potential biomarker for the diagnosis of liver fibrosis. The results of ROC analysis revealed that liver cMTO1 had a significant diagnostic value for liver fibrosis in patients with cirrhosis. However, the sample size is relatively small and studies with larger sample sizes are warranted to validate the efficacy of this marker. It is well known that blood-based biomarkers are non-invasive and more easily accepted by patients. Serum non-coding RNAs such as miRNAs and lncRNAs have been shown to be novel biomarkers for diagnosis of diseases (Li et al., 2011). Due to highly conserved sequences and a high degree of stability, circRNAs are superior to miRNAs and lncRNAs in the diagnosis of human diseases. Therefore, it is interesting that whether serum cMTO1 is a promising biomarker in liver fibrosis. Further studies should be performed to prove it.

**FIGURE 8:**
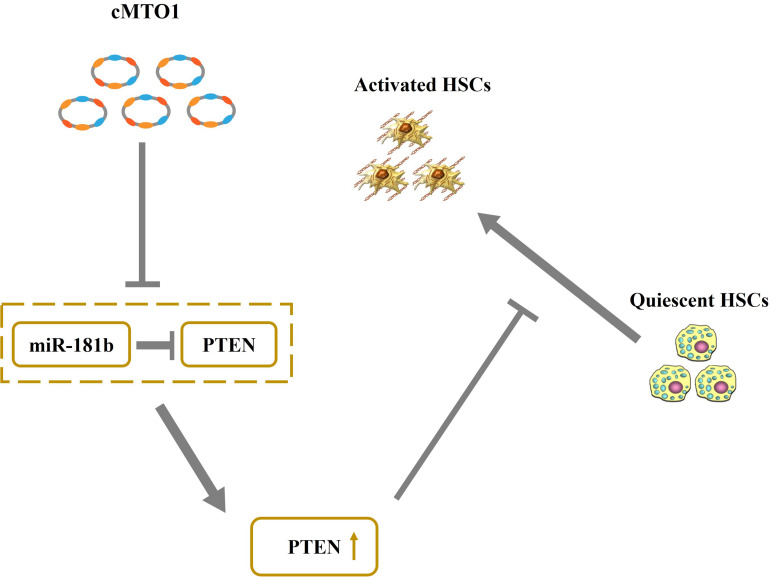
Schematic representation of a working model by which cMTO1 promotes PTEN expression in HSCs.

cMTO1, down-regulated in HCC, has been reported to serve as a tumor suppressor (Han et al., 2017). In this study, cMTO1 was reduced in human liver cirrhotic tissues, similar with the previous results. In line with it, down-regulation of cMTO1 expression was confirmed in CCl_4_-induced mice. Moreover, there was a significant decrease in cMTO1 expression in isolated primary HSCs with time in culture. It is known that HSC activation is featured with excessive collagens, increased α-SMA level and enhanced cell proliferation. Herein, we found that cMTO1 promoted the suppression of HSC activation. Restoring of cMTO1 led to the suppression of HSC proliferation. cMTO1 induced a reduction in the α-SMA expression as well as type I collagen. Taken together, cMTO1 plays an anti-fibrotic role in liver fibrosis. Furthermore, it was found that the effects of cMTO1 on HSC activation were blocked down by miR-181b-5p or loss of PTEN, suggesting that cMTO1 inhibits HSC activation, at least in part, through miR-181b-5p/PTEN axis (Figure 8). To the best of our knowledge, this is the first study to show that cMTO1 contributes to the suppression of liver fibrosis progression via miR-181b-5p-mediated PTEN expression.

Recently, circRNAs have been reported to regulate gene expression via interacting with miRNAs or other molecules (Hansen et al., 2013). Particularly, circRNAs are shown to contribute to cancer progression via regulating tumor-related genes by sponging miRNAs (Hansen et al., 2013). For instance, cir-ITCH acts as a tumor suppressor in esophageal squamous cell carcinoma via sponging miR-7, miR-17, and miR-214 (Li F. et al., 2015). In addition, circRNAs have been reported to be involved in the progression of fibrotic diseases such as cardiac fibrosis and pulmonary fibrosis (Li et al., 2019; Gu et al., 2020). Herein, we found that cMTO1 participated in the progression of liver fibrosis. cMTO1 was predominantly expressed in the cytoplasm. Bioinformatic analysis predicted that cMTO1 could bind to many miRNAs. Among these predicted miRNAs, miR-181b-5p reduced the luciferase reporter activity by at least 50%, whereas others not. Moreover, the pull-down assay showed that there was a direct interaction between cMTO1 and miR-181b-5p, which was further confirmed by double-FISH analysis. Interestingly, cMTO1 and miR-181b-5p can not be digested by each other. Of note, the anti-fibrotic effects of cMTO1 on HSC activation were almost blocked down by miR-181b-5p. Combined with these, we revealed that cMTO1 inhibits HSC activation, at least in part, via sponging miR-181b-5p.

miR-181b is described as an oncogenic miRNA in cancers (Wang et al., 2010). Recently, up-regulation of miR-181b has been shown to accelerate the progression of liver fibrosis. For instance, Wang et al. reported that miR-181b promotes HSCs proliferation by targeting p27 (Wang et al., 2012). miR-181b acts as a pro-fibrotic miRNA in liver fibrosis. Increasing evidence suggests that PTEN, suppressing liver fibrosis progression, is a target of miR-181b (Zheng et al., 2015). It was found that cMTO1 induced an increase in PTEN protein. Loss of PTEN resulted in the suppression of the effects of cMTO1 on HSC activation. In sum, our results suggest that cMTO1 contributes to the suppression of HSC activation, in partly, via miR-181b/PTEN axis.

In conclusion, we demonstrate that cMTO1 inhibits HSC activation, at least in part, through miR-181b-5p-mediated PTEN expression. Our results also provide a new insight of the anti-fibrotic roles of circRNAs in liver fibrosis.

## Data Availability Statement

All datasets generated for this study are included in the article/Supplementary Material.

## Ethics Statement

The studies involving human participants were reviewed and approved by the Ethics Committee of the First Affiliated Hospital of Wenzhou Medical University. The patients/participants provided their written informed consent to participate in this study. The animal study was reviewed and approved by the Ethics Committee of the Wenzhou Medical University. Written informed consent was obtained from the individual(s) for the publication of any potentially identifiable images or data included in this article.

## Author Contributions

JZ conceived and designed the work. PD acquired the biological samples and analyzed the data. All authors conducted the experiments, drafted the work, reviewed, and approved the final manuscript.

## Conflict of Interest

The authors declare that the research was conducted in the absence of any commercial or financial relationships that could be construed as a potential conflict of interest. The reviewer ZL declared a shared affiliation with the authors to the handling editor at the time of review.
