# Supplementary material for: Circular RNA cMTO1 Promotes PTEN Expression Through Sponging miR-181b-5p in Liver Fibrosis
Source: Front Cell Dev Biol. 2020 Jul 31;8:714. doi: 10.3389/fcell.2020.00714 (PMC7413143; doi:10.3389/fcell.2020.00714)

**Fig.S1 The role of miR-181b in PTEN-dependent HSC activation.** Primary 1-day-old HSCs were transfected with miR-181b-5p inhibitor for 48 h. (A) PTEN protein expression. (B) α-SMA mRNA. (C) Col1A1 mRNA. Each value is the mean ± SD of three experiments. ^*^*P*<0.05 compared with the control.


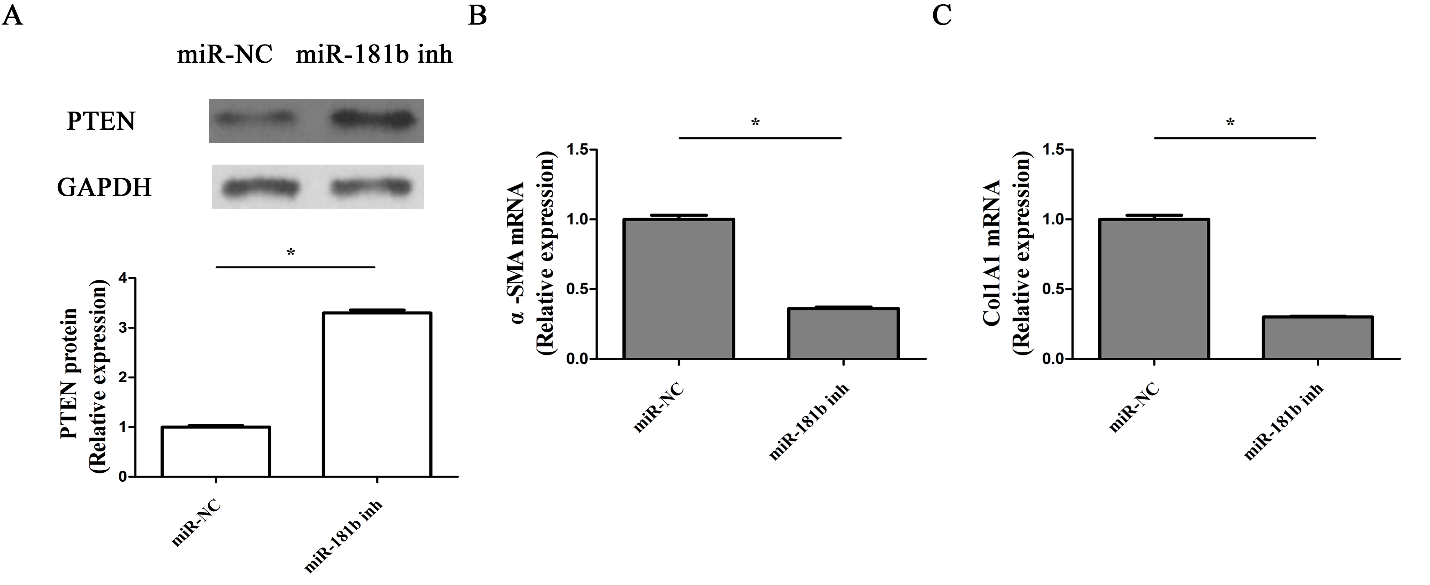

Supplement: Supplementary file 1 [file Data_Sheet_1.docx]
